# Case Report: Deciphering a *de novo* complex chromosomal rearrangement causing premature ovarian insufficiency, short stature, and mild intellectual disability using long-read sequencing

**DOI:** 10.3389/fgene.2026.1811784

**Published:** 2026-06-09

**Authors:** Qingqiu Cheng, Qi Peng, Fen Lv, Xiaomei Zeng, Xiaowen Chen, Siping Li, Xiaomei Lu

**Affiliations:** 1 Laboratory Department, Dongguan Children’s Hospital, Dongguan, Guangdong, China; 2 Department of Medical and Molecular Genetics, Dongguan Institute of Pediatrics, Dongguan, Guangdong, China; 3 Key Laboratory for Children’s Genetics and Infectious Diseases of Dongguan, Dongguan, Guangdong, China

**Keywords:** complex chromosomal rearrangement, gene disruption, genotype-phenotype correlation, long-read sequencing, position effect, premature ovarian insufficiency, X-inactivation

## Abstract

**Objective:**

Premature ovarian insufficiency (POI) with short stature and mild intellectual disability can have diverse genetic etiologies. We aimed to decipher the genetic basis of this complex phenotype in a 26-year-old female with a karyotype lacking aneuploidy.

**Design and Methods:**

This is a case study integrated with comprehensive genetic analyses. After standard techniques (karyotyping, CNV-seq, WES) failed to yield a diagnosis, Oxford Nanopore long-read sequencing was employed to map chromosomal breakpoints at single-base resolution. X-inactivation (XCI) analysis was also performed.

**Results:**

Long-read sequencing refined the karyotype to 46,X,t(X; 3;8) (q25; q21p21; p21),t(17; 22)(q21.2; q13) and identified direct disruptions of TAFA5, LARS2, and MYLK. XCI analysis demonstrated highly skewed XCI (5.35%), indicating preferential inactivation of the structurally normal X chromosome.

**Conclusion:**

We propose that highly skewed XCI is the primary driver of the patient’s POI and short stature, while the three disrupted genes may serve as modifying factors. This study underscores the value of long-read sequencing in resolving CCRs and the importance of XCI analysis in female patients with X-chromosome rearrangements.

## Introduction

Premature ovarian insufficiency (POI) affects approximately 1% of women and is characterized by loss of ovarian function before age 40. Although POI can occur together with short stature and mild neurodevelopmental abnormalities, the underlying genetic causes often remain unclear. In some patients with ostensibly “balanced” chromosomal translocations, cryptic imbalances or complex rearrangements may explain the clinical presentation (Stochholm et al., 2006).

Complex chromosomal rearrangements (CCRs) are defined as structural abnormalities involving at least three chromosomes and three or more breakpoints (Schuy et al., 2022). CCRs are rare, but when they occur, they can lead to significant clinical consequences due to gene dosage imbalance or position effects. Here we describe a patient with a *de novo* CCR involving five chromosomes, who presented with POI, short stature, and mild intellectual disability. To understand the genotype-phenotype relationship, we performed conventional karyotyping, CNV-seq, WES, Oxford Nanopore long-read sequencing, and X-inactivation analysis.

## Case presentation

The patient is a 26-year-old female who presented to our gynecology department in May 2021 with the chief complaint of oligomenorrhea. She was born full-term with a birth weight of 2.3 kg. Since childhood, she exhibited mild intellectual and motor developmental delay, short stature, and short fingers. Menarche occurred at age 14, followed by progressive oligomenorrhea leading to secondary amenorrhea. At age 18, she began hormone replacement therapy due to 14 months of amenorrhea, exhibiting treatment dependence (cessation of menses upon discontinuation). Previous external diagnoses included “central amenorrhea” and “hyperthyroidism,” and insulin resistance was clinically confirmed at age 19.

Physical examination revealed: bilateral underdeveloped breasts, normal perineal development, pubic hair presence, and acanthosis nigricans on both thighs.

**FIGURE 1:**
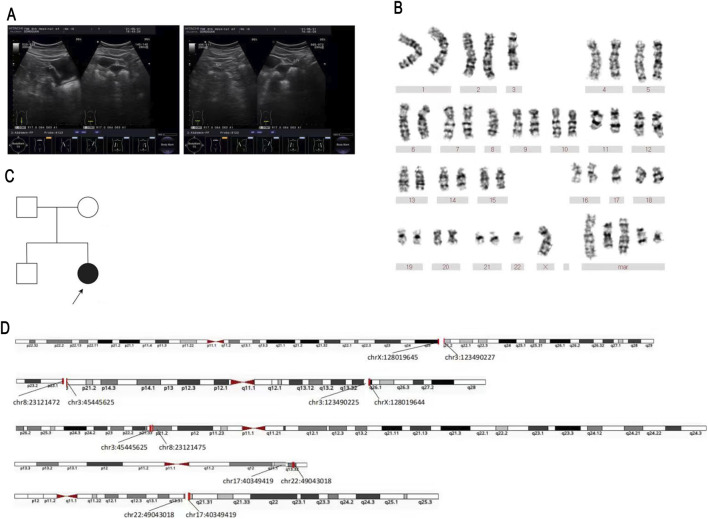
Clinical imaging and cytogenetic characterization of the patient. **(A)** Pelvic ultrasound imaging. Sagittal views demonstrate a markedly hypoplastic uterus. Bilateral ovaries exhibit significantly reduced volumes with no visible antral follicles. **(B)** Representative G-banded karyotype of the patient. The karyotype reveals the derivative chromosomes involved in the complex rearrangement. **(C)** Family pedigree. The pedigree chart illustrates the proband (solid circle, indicated by the arrow) and unaffected family members, confirming the *de novo* occurrence of the chromosomal rearrangement. **(D)** Schematic reconstruction of the complex chromosomal rearrangement. An integrative linear visualization of the derivative chromosomes and their precise breakpoints, combining G-banding karyotype findings with Oxford Nanopore long-read sequencing data.

Pelvic color Doppler ultrasound confirmed severe hypoplasia of the reproductive system: a markedly small uterus (volume 20 × 21 × 13 mm, corpus-to-cervix ratio 1:1, non-visualized endometrium) and bilaterally reduced ovarian volumes (left: 14 × 17 × 10 mm; right: 17 × 16 × 10 mm) without visible antral follicles (Figure 1A), consistent with premature ovarian insufficiency (POI).

Laboratory examinations further revealed the patient’s complex endocrine and metabolic abnormalities (Table 1). Key findings included: undetectably low anti-Müllerian hormone (AMH <0.01 ng/mL)), indicating depleted ovarian reserve; thyroid dysfunction (elevated TSH with normal FT3/FT4, consistent with subclinical hypothyroidism; prior history of hyperthyroidism); severe mixed dyslipidemia, hyperuricemia, and mild thrombocytosis.

**TABLE 1:** Major abnormal laboratory findings of the patient.

Test category	Test item	Patient’s result	Reference range
Reproduction & endocrinology	Anti-müllerian hormone (AMH)	0.01 ng/mL ↓	2.06–6.98 ng/mL
Thyroid function	Thyroid-stimulating hormone (TSH)	7.47 mIU/L ↑	0.27–4.2 mIU/L
	Free T3 (FT3)	4.89 pmol/L	3.1–6.8 pmol/L*
	Free T4 (FT4)	17.55 pmol/L	12–22 pmol/L
Metabolism & biochemistry	Total cholesterol (TC)	5.80 mmol/L ↑	≤5.17 mmol/L
	Triglycerides (TG)	2.31 mmol/L ↑	0.7–1.70 mmol/L
	Low-density lipoprotein cholesterol (LDL-C)	3.96 mmol/L ↑	2.07–3.10 mmol/L
	Uric acid (UA)	494 μmol/L ↑	179–390 μmol/L
Hematology	Platelet count (PLT)	373 × 10^9^/L ↑	125–350 × 10^9^/L

a, ↑/↓ indicate that the testing value is above or below the reference range, respectively. b, This table only lists abnormal indicators. The reference ranges are based on the Laboratory’s standard operation procedure.

In summary, the patient’s clinical, imaging, and laboratory profiles delineate a multisystem disorder centered on POI and sexual infantilism, with concomitant involvement of thyroid, glucose, and lipid metabolism. Karyotyping and long-read sequencing were deployed to resolve the genetic etiology of this case, identifying a first-reported *de novo* complex rearrangement and establishing a foundation for genetic counseling.

## Materials and methods

### G-banding karyotype analysis

Peripheral blood lymphocytes were cultured and harvested following standard procedures. Metaphase chromosomes were G-banded and analyzed under a light microscope. At least 20 metaphase spreads were analyzed. Karyotypes were described according to the International System for Human Cytogenomic Nomenclature (ISCN), with an analytical resolution of 320–400 bands.

### Low-depth whole-genome copy number variation sequencing (CNV-seq)

Genomic DNA was sequenced on the BGISEQ-500 platform using a commercial low-depth whole-genome sequencing kit (depth ∼0.1×, bin size 100 kb). CNV calling was performed with PSCC software. The reference genome was GRCh37/hg19. CNVs larger than 100 kb were reported.

### Whole-exome sequencing (WES)

Trio-based WES (proband and parents) was performed using a standard exome capture kit on an Illumina NovaSeq 6000 platform. Mean coverage was 168×, with 99.6% of targets covered at ≥20×. Reads were aligned to GRCh37/hg19; variants were called with GATK4 and annotated with ANNOVAR. Variants were filtered against gnomAD, ClinVar, and in-house databases.

### Oxford Nanopore long-read sequencing

High-molecular-weight genomic DNA was randomly sheared, and libraries were prepared using the SQK-LSK110 kit. Sequencing was performed on PromethION R9.4 flow cells, yielding whole-genome data with ∼15.2X mean coverage and an N50 of 28.8 kb.

Base calling was performed with Guppy (v0.3.0). Structural variant analysis was conducted via two independent pipelines: 1) LAST alignment followed by dnarrange analysis; 2) minimap2 alignment and variant calling with cuteSV (v2.1.1). All candidate breakpoints were manually verified using IGV.

### X-inactivation (XCI) analysis

XCI analysis was performed using the HUMARA method with the XCIFiler kit (fluorescent PCR-capillary electrophoresis, JingZhun Bio, Shanghai) following the manufacturer’s instructions. The kit contains four polymorphic genetic markers on the X chromosome (XpB, XpA, XpC, and XqA, with XqA being the AR locus). DNA was digested with HpaII prior to PCR amplification.

The XCI ratio was calculated as (D1/D2)/(U1/U2), where D and U represent peak heights after and before digestion, respectively. Products were separated by capillary electrophoresis. Skewed XCI was defined as a ratio <25%, and extremely skewed as <10%.

## Results

### Chromosome karyotype analysis of peripheral blood in the family

The patient’s karyotype was 46,X,t(X; 3;8)(q26; q21p21; p21),t(17; 22)(q12; q13) (Figure 1B). Long-read sequencing refined the Xq26 breakpoint to Xq25 (chrX:128,019,645/644) and the 17q12 breakpoint to 17q21.2 (chr17:40,349,419). This karyotype, the first reported in China, has been deposited in the Chinese Human Chromosome Abnormality Karyotype Database (No. 4953). Parental and elder brother’s karyotypes were normal, confirming the *de novo* origin of the CCR (Figure 1C).

### CNV-seq and WES findings

Both CNV-seq and WES identified no pathogenic copy number variations or pathogenic single-nucleotide variants/indels that could explain the complex phenotype.

### Oxford nanopore sequencing analysis

Nanopore sequencing resolved the complex rearrangement at single-base resolution. All six breakpoints were mapped (Figure 1D). The results refined the X-chromosome breakpoint from the karyotype-inferred Xq26 to Xq25 (chrX:128,019,645/644, 0.68 Mb distant) and the chromosome 17 breakpoint from 17q12 to 17q21.2 (chr17:40,349,419, 2.25 Mb distant). The rearrangement comprises a triple translocation involving chromosomes X, 3, and 8, and an independent t(17; 22) reciprocal translocation. Critically, three breakpoints at 22q13.3, 3p21.3, and 3q21.1 directly disrupt the *TAFA5, LARS2, and MYLK* genes, respectively (Table 2).

**TABLE 2:** Breakpoint analysis of the complex chromosomal rearrangement by Oxford Nanopore long-read sequencing.

Derivative chromosome	Involved chromosomal segments	Breakpoint coordinates (hg19)	Corresponding gene/Region
der(X)	3q21.1→Xq25	chr3:123,490,227 chrX:128,019,645	3q21.1 (Intragenic, *MYLK*) Xq25 (POI critical region)
der(3)	8p21.3→3p21.3 Xq25→3q21.1	chr8:23,121,472 chr3:45,445,625 chrX:128,019,644 chr3:123,490,225	8p21.3 3p21.3 (intragenic, *LARS2*) Xq25 (POI critical Region) 3q21.1 (intragenic, *MYLK*)
der(8)	3p21.3→8p21.3	chr3:45,445,625 chr8:23,121,475	3p21.3 (Intragenic, *LARS2*) 8p21.3
der(17)	17q21.2→22q13.3	chr17:40,349,419	17q21.2
der(22)	22q13.3→17q21.2	chr22:49,043,018	22q13.3 (Intragenic, *TAFA5*)

1. This table identifies Breakpoints of Complex chromosomal rearrangements using Oxford Nanopore long-read sequencing technology, with hg19 as the reference genome. 2. The symbol “→” indicates the Translocation direction of Chromosomal segments. 3. Gene region annotation is based on alignment of Breakpoints with the RefSeq gene database; “Within gene” indicates that the Breakpoint is directly located within the Coding or regulatory sequence of the gene.

### X-inactivation analysis

**FIGURE 2:**
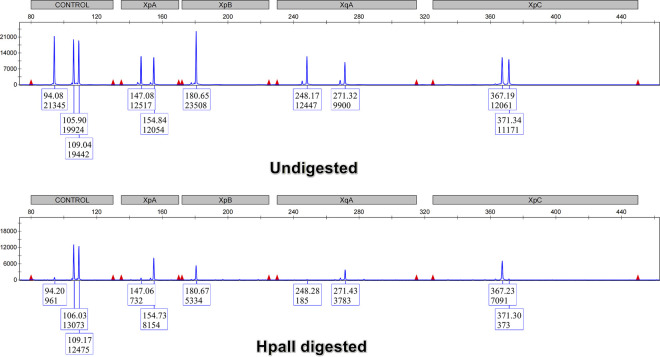
X-chromosome inactivation (XCI) analysis via the HUMARA assay. The upper panel displays the undigested control, showing two distinct peaks that correspond to the two maternal and paternal androgen receptor (AR) alleles. The lower panel presents the sample following digestion with the methylation-sensitive restriction enzyme HpaII. The amplification of one allele is markedly reduced (peak height ratio of 5.35%), indicating an extremely skewed X-chromosome inactivation pattern in the patient.

XCI analysis showed highly skewed inactivation (mean ratio 5.35%). Individual ratios for XpA, XqA, and XpC were 7.48%, 3.74%, and 5.37% (XpB not informative). After HpaII digestion, one allele was markedly reduced (Figure 2), indicating that >94% of cells inactivate the same X chromosome.

## Discussion

We report the first identification in China of a *de novo* complex chromosomal rearrangement (CCR) with a refined karyotype of 46,X,t(X; 3;8)(q25; q21p21; p21),t(17; 22)(q21.2; q13) determined by Oxford Nanopore long-read sequencing. The patient presented with premature ovarian insufficiency (POI), short stature, and mild intellectual disability. CNV-seq and WES did not detect any pathogenic copy number variants or single-nucleotide variants.

X-inactivation analysis revealed highly skewed XCI, with a mean ratio of 5.35% (individual ratios: XpA 7.48%, XqA 3.74%, XpC 5.37%; XpB not informative). Thus, >94% of peripheral blood cells inactivate the same X chromosome (Figure 2). In females carrying an X-autosome translocation, the structurally normal X is preferentially inactivated to maintain dosage balance (Therman and Pätau, 1974; Kalz-Füller et al., 1999). The most parsimonious interpretation is that the patient’s normal X is inactivated, leaving the derivative X (der(X)) active. Long-read sequencing refined the breakpoint to Xq25 (chrX:128,019,645/644), which lies within the well-established POI critical region Xq13–Xq26 (Mumm et al., 2001; Powell et al., 1994); haploinsufficiency of genes in this region is a recognized cause of ovarian failure and short stature. The original G-banding assignment at Xq26 is at the distal edge of this region and has not been consistently implicated. Thus, the precise mapping to Xq25 provides a coherent genotype–phenotype correlation. We therefore propose that skewed XCI is the primary driver of the patient’s core phenotype. The possibility of non-skewed XCI is therefore ruled out in this patient. This case also illustrates that karyotype-based breakpoint assignment requires high-resolution molecular confirmation—such as long-read sequencing—when precise genotype–phenotype correlations are sought (Pettersson et al., 2024).

Three autosomal breaks were identified, disrupting *TAFA5, LARS2*, and *MYLK*. However, several lines of evidence argue against their being primary causes. *TAFA5* has no established disease association in humans and its proposed role rests solely on mouse models (Shahapal et al., 2025); *LARS2*-related Perrault syndrome is recessive, and monoallelic disruption is not pathogenic (Adam et al., 2025); *MYLK* has no known clinical correlate (Lazar and Garcia, 1999; Jin et al., 2023). Given the clear X-linked mechanism (skewed XCI with breakpoint in the POI critical region), the contribution of these autosomal breaks—if any—is likely minor and modifying at best. The 8p21.3 and 17q21.2 breakpoints, which do not disrupt coding sequences, are even less likely to be relevant.

Several limitations should be acknowledged. First, CNV-seq and WES have inherent limitations in detecting small or cryptic copy number variants; therefore, the presence of such imbalances cannot be completely ruled out. Second, XCI analysis was performed only on peripheral blood, which may not fully reflect tissue-specific patterns (e.g., in ovaries or brain). Third, functional validation of the disrupted genes is lacking. Finally, this is a single case, and the findings require confirmation in additional patients. In addition, mechanistic studies such as microhomology analysis at the breakpoints were not performed due to the case report scope, but they are warranted for deeper insight into the rearrangement mechanism.

In summary, the complex phenotype of POI, short stature, and mild intellectual disability in this patient is best explained by a two-layer model. The primary layer is highly skewed XCI leading to functional haploinsufficiency of X-linked genes at Xq25, which drives the core features. The secondary layer consists of the three autosomal gene disruptions (*TAFA5, LARS2, MYLK*), which may serve as minor modifiers. This refined model acknowledges the dominant role of the X-chromosome abnormality while preserving the potential relevance of the disrupted autosomal genes.

## Conclusion

By integrating long-read sequencing and X-inactivation analysis, this study deciphered the genetic basis of a complex phenotype (premature ovarian insufficiency, short stature, and mild intellectual disability) caused by a *de novo* complex chromosomal rearrangement. We report for the first time a CCR with karyotype 46,X,t(X; 3;8)(q25; q21p21; p21),t(17; 22)(q21.2; q13). We propose a two-layer model: highly skewed X-inactivation (5.35%) as the primary driver of the core phenotype, and disruptions of *TAFA5*, *LARS2*, and *MYLK* as potential minor modifiers. This work highlights the value of long-read sequencing for precise breakpoint mapping and the necessity of X-inactivation analysis in female patients with X-chromosome rearrangements.

## Data Availability

The original contributions presented in the study are included in the article/supplementary material, further inquiries can be directed to the corresponding author.
